# Association of Social Support and Self-Care Beverage Consumption in Adults With Type 2 Diabetes From the Southwest of Mexico

**DOI:** 10.1155/nrp/2262033

**Published:** 2025-11-21

**Authors:** Geu Mendoza-Catalan, Julieta Ángel-García, Angélica S. Jiménez-Osorio, Diego Estrada-Luna, Olga Rocío Flores-Chávez, Carlos Ángel Gallardo-Casas

**Affiliations:** ^1^School of Nursing, Autonomous University of the State of Hidalgo, San Agustín Tlaxiaca, Hidalgo, Mexico; ^2^System of State Universities of Oaxaca, Santa Lucia del Camino, Oaxaca, Mexico

**Keywords:** beverage consumption, self-care, social support, type 2 diabetes

## Abstract

**Background:**

Social support in diabetes is considered a key element for individuals to self-care adherence. However, there is limited knowledge about social support and its impact on the self-care beverage consumption in adults with type 2 diabetes (T2D). The aim of this study is to analyze the association between social support and self-care related to beverages consumption in Mexican adults with T2D.

**Methods:**

This cross-sectional study included adults aged 20 and older with T2D from community health centers. The Social Support Scale for Self-Care in T2D questionnaire was used, and participants were asked about 15 types of beverages according to recommendations for the Mexican population.

**Results:**

A total of 246 participants with T2D were included. Social support for self-care was associated with increased consumption of unsweetened coffee (*r* = 0.146, *p* < 0.01), fruit juice (*r* = 0.102, *p* < 0.05), and low-fat milk (*r* = 0.117, *p* < 0.05) and a reduction in whole milk consumption (*r* = −0.108, *p* < 0.05). Men reported higher consumption of fruit juices and soft drinks, as well as generally higher consumption of beverages from categories 5 (whole milk, fruit juices, sports drinks, beer, wine, and spirits, *p* < 0.01) and 6 (cola, industrialized fruit juices and teas, sweetened coffee, and tea, *p* < 0.01), which are less recommended.

**Conclusion:**

Social support was associated with a higher consumption of fruit juice, low-fat milk, and unsweetened coffee and a lower consumption of whole milk. It is important to integrate educational programs to improve self-care regarding beverage consumption in individuals with T2D.

## 1. Introduction

Diabetes is a widespread and complex chronic disease that is a major concern due to its prevalence, mortality rates, and healthcare costs in worldwide. Globally, 10.5% of the population has diabetes, with type 2 diabetes (T2D) being the most prevalent, and 80% of the cases occurring in middle- and low-income countries [1]. Mexico ranks 7th in the world in terms of the number of cases [1], with a prevalence of 18.3%, and it is the second leading cause of national mortality [2]. It is well recognized that ongoing diabetes self-management education and support are critical to prevent acute complications and empower people to improve glycemic control [3].

According to the Association of Diabetes Care and Education Specialists, individuals need to engage in seven self-care activities, which are healthy coping, healthy eating, being active, taking medication, monitoring, reducing risk, and problem solving [3]. Among these activities, adhering to a healthy diet has been considered the most challenging due to cultural factors, costs, and access to unhealthy foods [4, 5]. To control calorie intake, it is recommended to avoid sugary beverages, and instead opt for water, low-calorie or calorie-free drinks [3, 6]. A study in the southwest of Mexico reported that among people with T2D, 41.6% consume soft drinks, 34.7% consume coffee with sugar, 31.8% drink natural fruit juices, and 21% consume industrialized juices [7], all of which are not recommended [8]. However, people consume these beverages to feel more energetic at work [9]. Individuals with T2D who consume sugary beverages are mostly men, smokers, under 40 years old, and those who have been diagnosed with T2D for less than 5 years [7, 10, 11].

Self-care beverage consumption is the voluntary and conscious intake of liquids whose nutritional characteristics (sugar content, calories, nutrient value, and hydration capacity) promote overall health, prevent disease, and support well-being, in contrast to beverages with a high energy content, high added sugar content, or low nutritional value [8]. It has been reported that individuals with T2D who consume sugary beverages have higher body mass index (BMI), low density lipoprotein–cholesterol, and glycated hemoglobin (HbA1c) levels. This consumption also increases their risk of mortality by 17% [10, 12–14]. On the other hand, the consumption of tea, coffee, diet beverages, beer, and wine is linked to improve glycemic control and reduce mortality. Additionally, the consumption of low-fat milk can lead to decreased body fat percentage and BMI [12, 15–18].

There are several psychosocial factors that can influence self-care in individuals with T2D, one of the most important being social support. Social support in diabetes care is considered a key element for individuals to make significant lifestyle changes and adhere to medical recommendations [19]. Social support is defined as any help received by individuals with T2D, including tangible, instrumental, financial, or informational support, to improve their self-care [20]. According to various studies, social support enhances self-care, medication adherence, quality of life, improves glycemic control, and reduces the risk of mortality [21–25]. Nevertheless, there is also evidence that social support also has negative effects, such as promoting self-medication, lack of pharmacological adherence by seeking alternative therapies, and influencing incorrect lifestyle choices (alcohol and tobacco consumption and poor dietary habits) during social events [26–30].

Furthermore, to theoretically frame the relationship between social support and health behaviors in T2D, this study is guided by the Specific Theory regarding family behavior of people who have T2D [26]. This theory posits that social dynamics and the specific types of support provided by the social network are crucial determinants in the self-care practices adopted by individuals with T2D, which can include self-care related to beverage consumption. To the best of the present researchers' knowledge, no study has specifically examined the relationship between social support and self-care related to beverage consumption in individuals with T2D from the southwest of Mexico. Therefore, the results of this study may provide valuable knowledge for evidence-based nursing decision-making and the development of public health strategies aimed at strengthening social support programs and reducing the consumption of nonrecommended beverages in individuals with T2D.

Due to the health issue that T2D represents for society and healthcare systems, this research was conducted to analyze the relationship between social support and self-care related to beverage consumption in Mexican adults with T2D. The specific objectives were to compare beverage consumption by sex and to explore the association between beverage intake and participant characteristics.

## 2. Materials and Methods

### 2.1. Study Population

A cross-sectional study was conducted with adults over 20 years of age with a diagnosis of T2D from community health centers in the State of Oaxaca, Mexico. The sample size was calculated using the G^∗^ Power 3.1.9.7 program for a correlation analysis, with an expected effect size of 0.20 [22–24], a power of 0.90, and a 5% alpha error, resulting in a required sample size of 258 participants. A nonprobabilistic convenience sampling method was used, and participants were recruited from December 2018 to February 2019. Inclusion criteria comprised individuals with a previous T2D diagnosis of more than one year who were active patients attending their follow-up appointments at the health centers. We excluded individuals with mental health issues or those undergoing psychiatric treatment, those with lower limb amputations, diabetes-related complications such as nephropathy (requiring dialysis or hemodialysis), cardiopathy, and severe diabetic retinopathy (with vision loss), as well as those who had undergone surgery in the last 6 months.

### 2.2. Questionnaires

#### 2.2.1. Social Support in Diabetes

Social support was measured using the Social Support Scale for Self-Care in T2D [31] in its version adapted for the Mexican population [32]. The instrument consists of 30 items focused on support for diet, exercise, foot care, self-monitoring blood glucose, and smoking cessation. Each item is rated on a 5-point Likert scale, from 1 (*never*) to 5 (*always*). Higher scores indicate greater social support in self-care for T2D. The original author [31] and the Mexican adaptation [32] both reported a Cronbach's alpha of 0.94. The Cronbach's alpha obtained in this study was 0.95, which is considered as excellent reliability.

#### 2.2.2. Self-Care Beverage Consumption

Self-care in beverage consumption was guided by the Beverage Recommendations for the Mexican Population, which classifies beverages into six categories [8]. Category 1: water (natural or mineral water); category 2: low-fat milk (semiskimmed or skimmed); category 3: unsweetened coffee and/or tea; category 4: noncaloric beverages with artificial sweeteners (diet or light beverages); category 5: beverages caloric and limited health benefits (whole milk, fruit juices, sports drinks, beer, wine, and spirits); and category 6: sugary beverages with low nutrient content (cola, industrialized fruit juices and teas, sweetened coffee, and tea). Participants were asked individually about their beverage intake frequency in a typical week and the amount of beverage per occasion (one serving was measured as 240 mL). Weekly consumption was calculated for each beverage individually, and an average consumption was obtained for beverages in categories 2, 5, and 6, which include various types of drinks.

#### 2.2.3. Covariates

Age, sex, education level, marital status, years since T2D diagnosis, hours of sleep, working hours, smoking, previous diagnosis of hypertension, and BMI were included as covariables.

### 2.3. Data Collection

Data collection was conducted through community health centers in the coastal region of the state of Oaxaca. At each center, individuals in the waiting room were identified to determine who had a diagnosis of T2D. Those identified as participants were invited to join the study. Interested participants were then invited to move to a private area within the health center, either before or after their medical appointment (as per the participant's preference). Each participant was given and read the informed consent form, questions were answered, and participants were asked to sign the document and receive a copy. Subsequently, the questionnaires were administered using a face-to-face interview format. The study was approved by the research ethics and research committees of the School of Nursing of the Autonomous University of Baja California with registration number 0192088.

### 2.4. Statistical Analysis

The questionnaires were entered and analyzed using SPSS v. 26. For categorical variables, frequencies and percentages were used, while for continuous variables, mean, median, and standard deviation (SD) were employed. According to the Kolmogorov–Smirnov normality test, the variables were identified as having a nonparametric distribution. Subsequently, Spearman's correlation analysis was conducted, including general and dimension-specific social support, consumption of different types of beverages, and consumption from categories 2, 5, and 6. Categories 1 (water), 3 (unsweetened coffee), and 4 (diet drinks) were treated as a single type of beverage. The correlation analysis also included the following covariates: age, number of years of schooling, years since diagnosis with T2D, BMI, hours of sleep, and hours of work. Finally, the Mann–Whitney *U* test was used to identify differences in beverage consumption between men and women.

## 3. Results

### 3.1. Sample Outcomes

A total of 246 participants were involved. The average age was 53.3 years (SD = 9.3), with the majority being women and married. The average number of years of schooling was 5.2 (SD = 4.0), sleep was 6.4 h (SD = 3.2), and average years since T2D diagnosis was 9.0 years (SD = 6.6). Among the participants, 28.5% had a diagnosis of hypertension, 12.2% were smokers, and 70.7% were classified as overweight/obesity. The detailed data are presented in Table 1.

### 3.2. Beverage Consumption by Sex

According to weekly beverage consumption, after water, unsweetened coffee was the second consumed beverage, followed by beer, sweetened coffee, sports/energy drinks, sweetened tea, whole milk, reduced fat milk, soft drinks, fruit juice, sweetened juices, diet beverages, low fat milk, and liquors (Table 2). When comparing by sex, it was observed that men consume more fruit juices and soft drinks, as well as higher consumption of beverages in categories 5 and 6.

### 3.3. Correlation of Social Support and Self-Care in Beverage Consumption

As shown in Table 3, overall social support was correlated with lower consumption of whole milk and higher consumption of fruit juice, reduced fat milk, and unsweetened coffee. Among the five dimensions of social support, smoking cessation support was correlated with the majority of beverages (9/14), followed by support for diet (8/14), support for glucose monitoring (7/14), support for foot care (5/14), and finally, support for exercise (2/14). According to the covariates, age was correlated with the consumption of soft drinks (*r* = −0.103, *p*=0.016) and beer (*r* = −0.129, *p*=0.003). Years of schooling was correlated with the consumption of soft drinks (*r* = 0.104, *p*=0.015), fruit juices (*r* = 0.151, *p*=0.001), unsweetened coffee (*r* = 0.094, *p*=0.029), and beer (*r* = 0.086, *p*=0.045). The years since T2D diagnosis was correlated with unsweetened coffee consumption (*r* = 0.127, *p*=0.007). Working hours were correlated with whole milk (*r* = −0.109, *p*=0.011), sweetened tea (*r* = 0.101, *p*=0.019), and sweetened coffee (*r* = 0.137, *p*=0.001). BMI was correlated with sweetened juices (*r* = 0.101, *p*=0.046), reduced fat milk (*r* = −0.172, *p*=0.001), and sweetened tea (*r* = 0.103, *p*=0.041). Sleep hours were related to the consumption of beer (*r* = −0.093, *p*=0.031) and liquors (*r* = −0.150, *p*=0.001), data not shown.

## 4. Discussion

The objective of this study was to analyze the relationship between social support and self-care related to beverage consumption in Mexican individuals with T2D. Our results highlight that perceived overall social support and its different dimensions were associated with an increase in the consumption of various recommended beverages and a decrease in nonrecommended beverages for the Mexican population.

In our study, the most consumed beverages by category were water, followed by categories 6 (sugary beverages with low nutrient content), 3 (unsweetened coffee and/or tea), 5 (whole milk, fruit juices, sports drinks, beer, wine, and spirits), 4 (noncaloric beverages with artificial sweeteners), and 2 (low-fat milk). This consumption pattern does not align with the recommended beverage consumption guidelines for the Mexican population [8] and individuals with T2D [6]. This implies that even after a diagnosis of the disease, individuals continue to consume such sugary beverages for various reasons. One explanation could be the addiction created by the consumption of these types of beverages during development and before T2D diagnosis [33, 34]. Additionally, in a study with Mayan subjects with T2D from rural communities from the south and east of México, it was reported that eliminating sugary beverages and inexpensive industrialized foods was perceived as difficult or impossible and the nonadherence to better diet regimen is influenced by recommendations misaligned with local culture [35]. The consumption of sugary beverages has been identified as a risk factor for impaired beta cell function, had higher HbA1c levels [36] and higher risk of BMI in Mexican T2D patients [37].

The consumption of low-calorie or diet beverages was also observed in this study and the inclusion of coffee and tea also was associated with cultural factors in the south of Mexico [38]. Although coffee and tea consumptions are generally associated with health [39] in a previous report in regions near to Oaxaca, Mexico, the coffee intake was not associated with the prevalence of self-reported T2D [40]. In addition, it has been reported that individuals who often consume beverages with low calories end up consuming more total fats, saturated fats, carbohydrates, and sugars through their food intake [41]. Also, the use of artificially sweetened beverages has been associated with metabolic syndrome [42], loss of beta cell function [43, 44], and higher risk of cardiovascular disease in T2D patients [45, 46]. Therefore, the education of the selection of beverages considered as healthy needs to be accompanied by the elimination of those beverages and foods who represent higher risk to T2D and its complications.

It has been reported that men and women with diabetes had differences in adherence to nutritional recommendations [47]. In this study, men had a higher frequency of fruit juice consumption than women, as well as beverages with limited health benefits and sugary beverages with low nutrient content. This responds to multifactorial causes influenced by education and perceptions. The reduction of sugar sweetened beverages is effective in women when they perceive a bad image-related physical appearance. Conversely, women had a negative attitude to this kind of beverage when they perceived muscle mass loss [48].

It has been widely recognized that social support plays a crucial role in promoting various self-care behaviors including exercise, medication adherence, glucose self-monitoring, and foot care [19]. Our results identified that overall social support and its dimensions (diet, self-monitoring, and foot care) display both positive and negative associations with different beverages choices, including both recommended and nonrecommended drinks. General social support has been associated with higher consumption of unsweetened coffee and low-fat milk and lower consumption of whole milk. In contrast, diet-specific support and self-monitoring of blood glucose are linked to reduced intake of soft drinks and beer. These findings may be explained by psychosocial mechanisms previously identified in the literature. Social support may facilitate disease coping by reducing stress and emotional distress [19, 21, 22] and also enables individuals to enhance their self-efficacy and empowerment in self-care, leading to better glycemic control [19, 22, 23]. This suggests that incorporating social support into these areas could contribute to improved adherence to recommended beverage intake. Therefore, it would be beneficial for individuals close to those with T2D—such as family, friends, and neighbors—to be educated about the importance of moderating the consumption of different types of beverages.

In contrast, the social support received for smoking cessation was associated with higher consumption of soft drinks, sweetened coffee, sports or energy drinks, and alcoholic beverages such as beer and liquors. Some previous studies have observed that, after quitting smoking, people often increase their intake of sugary foods and drinks [49–51]. This tendency might be explained by a search for pleasure or emotional compensation, as well as by the activation of dopaminergic reward circuits—mainly the mesolimbic and mesocortical pathways. These areas are responsible for dopamine release in the nucleus accumbens, a process linked to sensations of pleasure and reinforcement, but also to reduced impulse control and decision-making capacity [34]. As a result, greater consumption of sweet foods and beverages after smoking cessation has been associated with weight gain and, in people with diabetes, with difficulties in maintaining glycemic control [51, 52]. Therefore, smoking cessation treatments should ideally combine pharmacological support with behavioral and nutritional counseling to help manage withdrawal-related effects [52, 53].

It is important to consider that future programs aimed at social support to improve adherence to self-care in T2D should include components on recommendations for the consumption of different types of beverages and their future health implications. Additionally, such programs should provide information on strategies for quitting smoking and address withdrawal symptoms to prevent relapse into the consumption of sweetened beverages.

This study has several limitations. First, it was a cross-sectional study, which means that causal relationships cannot be determined. Second, the sampling was nonprobabilistic and based on convenience, which may limit the generalizability of the findings. Third, beverage consumption was self-reported, which may introduce recall bias. Future research should incorporate objective measures or dietary records and include key covariates—such as income level, food prices, and beverage availability—to better understand the contextual and socioeconomic factors influencing beverage choices among individuals with T2D. As strengths, this is the first study to evaluate social support and the consumption of various types of beverages in individuals with T2D. It provides insight into beverage consumption among the Mexican population with T2D and contributes to the knowledge base for future interventions in this area.

## 5. Conclusions

The consumption of nonrecommended beverages from category 5: beverages caloric and limited health benefits (whole milk, fruit juices, sports drinks, beer, wine, and spirits) and category 6: sugary beverages with low nutrient content (cola, industrialized fruit juices and teas, sweetened coffee, and tea) was high among individuals with T2D. Men were found to consume more fruit juices, soft drinks, and generally more beverages from categories 5 and 6. Social support was associated with increased consumption of recommended beverages and a reduction in nonrecommended beverages. The dimension of social support for smoking cessation was correlated with the consumption of a higher number of nonrecommended beverages, including soft drinks, sweetened coffee, sports drinks, and alcoholic beverages.

## Figures and Tables

**Table 1:** Description of participant characteristics.

Variables	*n*	%
Age M (SD)	53.3 (9.3)	
Sex		
Woman	174	70.7
Man	72	29.3
Marital status		
Single	40	16.3
Married	136	55.3
Common law	46	18.7
Other	34	13.8
Years of schooling M (SD)	5.2 (4.0)	
Sleep hours M (SD)	6.4 (3.2)	
Years T2D diagnosis M (SD)	9.0 (6.6)	
Smoker	30	12.2
Hypertension diagnosis	70	28.5
BMI		
Normal	72	29.3
Overweight	72	29.3
Obesity	102	41.4
Working hours M (SD)	45.1 (13.5)	
< 40 h	103	41.9
> 40 h	144	58.1

*Note:* M = median.

Abbreviations: BMI, Body Mass Index; SD, standard deviation.

**Table 2:** Comparison of beverage consumption by sex in individuals with T2D.

Variables	General		Women		Men	
	M (SD)	Mdn	M (SD)	Mdn	M (SD)	Mdn
Water	46.3 (32.7)	35.0	44.7 (30.5)	35.0	50.2 (36.9)	35.0
Fruit juice^∗^	2.7 (2.0)	2.5	2.5 (1.8)	2.0	3.2 (2.2)	2.7
Whole milk	3.5 (2.9)	2.5	3.5 (3.2)	2.2	3.6 (2.2)	2.5
Reduced fat milk	3.5 (2.3)	3.0	3.4 (2.3)	3.0	3.7 (2.2)	3.2
Low-fat milk	3.3 (1.0)	3.5	4.5 (3.0)	3.6	3.7 (3.1)	3.4
Sweetened juices	2.6 (1.7)	2.5	2.5 (1.6)	2.3	2.9 (1.7)	2.5
Soft drink^∗^	3.4 (2.5)	2.5	2.7 (2.1)	2.0	4.8 (3.9)	3.0
Diet beverages	2.5 (1.7)	2.0	2.4 (1.7)	2.1	2.8 (1.8)	2.0
Sweetened tea	4.0 (2.6)	3.7	3.9 (2.5)	3.9	4.5 (2.7)	4.3
Unsweetened coffee	6.1 (2.9)	6.2	6.1 (2.9)	6.1	6.4 (3.0)	6.5
Sweetened coffee	5.6 (3.3)	5.2	5.5 (3.3)	5.2	6.0 (3.2)	5.8
Beer	5.8 (4.1)	3.7	5.8 (4.2)	3.7	5.8 (4.1)	3.7
Liquors	1.6 (0.3)	1.5	1.5 (0.2)	1.5	1.6 (0.4)	1.6
Sports/energy drinks	4.1 (2.5)	3.0	4.1 (2.5)	3.0	4.0 (2.9)	3.0
Category 2	3.8 (2.6)	3.0	3.8 (2.7)	3.0	3.9 (2.4)	3.7
Category 5^∗∗^	4.3 (4.0)	3.0	4.0 (3.7)	2.8	5.2 (4.5)	3.7
Category 6^∗∗^	6.4 (5.4)	5.0	6.0 (4.8)	4.9	7.5 (6.4)	5.5

*Note:* M = media; Mdn = Median.

Abbreviation: SD, standard deviation.

^∗^ *p* < 0.05.

^∗∗^ *p* < 0.01.

**Table 3:** Correlation between social support and self-care in beverage consumption among adults with T2D.

Variables	Social support	Diet	Exercise	SMBG	Foot care	Smoking cessation
Water	0.040	0.082	0.042	0.032	0.005	−0.144^∗∗^
Fruit juice	0.102^∗^	0.103^∗^	0.074	0.105^∗^	0.060	0.046
Whole milk	−0.108^∗^	−0.129^∗∗^	0.002	−0.121^∗∗^	−0.103^∗^	0.027
Reduced fat milk	0.061	0.117^∗∗^	0.092^∗^	0.074	0.028	0.122^∗∗^
Low-fat milk	0.117^∗^	0.109^∗^	0.097^∗^	0.104^∗^	0.098^∗^	0.026
Sweetened juices	−0.014	0.037	−0.006	−0.048	−0.030	0.045
Soft drink	−0.070	−0.110^∗^	0.032	−0.106^∗^	−0.037	0.099^∗^
Diet beverages	−0.072	−0.041	−0.008	−0.083	−0.085^∗^	0.019
Sweetened tea	0.002	0.025	0.019	−0.073	0.013	0.141^∗∗^
Unsweetened coffee	0.146^∗∗^	0.122^∗∗^	0.029	0.129^∗∗^	0.159^∗∗^	0.098^∗^
Sweetened coffee	−0.072	0.001	−0.012	−0.115^∗∗^	−0.105^∗^	−0.084^∗^
Beer	−0.065	−0.108^∗^	0.004	−0.107^∗^	−0.069	0.134^∗∗^
Liquors	−0.018	−0.078	−0.023	−0.014	0.005	0.206^∗∗^
Sports/energy drinks	0.050	0.097^∗^	0.031	0.026	−0.018	0.102^∗^
Category 2	0.122^∗∗^	0.170^∗∗^	−0.038	0.127^∗∗^	0.084^∗^	0.092^∗^
Category 5	−0.042	−0.047	0.021	−0.045	−0.074	0.090^∗^
Category 6	−0.055	−0.008	0.008	−0.107^∗^	−0.078	0.131^∗∗^

Abbreviation: SMBG, self-monitoring of blood glucose.

^∗^ *p* < 0.05.

^∗∗^ *p* < 0.01.

## Data Availability

The data that support the findings of this study are available from the corresponding author upon reasonable request.
